# Synthetic polypeptides using a biologic as a reference medicinal product – the European landscape of regulatory approvals

**DOI:** 10.3389/fmed.2024.1335928

**Published:** 2024-04-12

**Authors:** Kevin Klein, Jens Heisterberg, Pieter Stolk

**Affiliations:** ^1^Exon Consultancy, Amsterdam, Netherlands; ^2^Novo Nordisk, Copenhagen, Denmark

**Keywords:** biosimilars, complex generics, synthetic polypeptide, hybrid application, non-biological complex drugs, therapeutic equivalence, liraglutide, teriparatide

## Abstract

Recent advances in synthetic drug manufacturing have introduced a new dynamic to the European regulatory system, with chemically synthesized polypeptide products using biological originator products as their reference medicine. Whereas biosimilars are subject to a dedicated regulatory framework in the EU, synthetically produced follow-on products are not eligible for assessment through this pathway, requiring approval via the traditional generic pathway under Article 10 (1), or via the hybrid pathway under Article 10 (3). This review presents an overview of recent developments in the field of synthetic peptides referencing biological originators in the EU. The use of different regulatory procedures can have potential implications for regulatory assessments, clinical practice and pharmacovigilance. As more complex synthetic products referencing recombinant originator products are expected in the coming years, this study promotes more transparency as well as global alignment about regulatory procedures for chemically synthesised products referencing biological originator products to ensure approval of safe and high-quality generics.

## Introduction

Recombinant DNA (rDNA) technology has transformed the pharmaceutical landscape over the past four decades. Since the approval of recombinant insulin (Humulin^®^) in 1988, hundreds of recombinant biologics have been approved in the European Union (EU) and are now responsible for almost half of all new medicine approvals annually. In 2005, the EU created a dedicated regulatory framework to ensure competition for biologics. The establishment of the biosimilar pathway through Article 10 (4) of Directive 2001/83/EC allowed for a robust evaluation of biological follow-on products (biosimilars) considering their inherent complexity (1). This led to the approval of the first biosimilar for recombinant somatropin in 2006 (Omnitrope^®^). While as of 2023, more than 80 biosimilar applications have successfully been reviewed and approved, a new challenge regarding generic competition has recently emerged in the polypeptide class (2).

**Figure 1:**
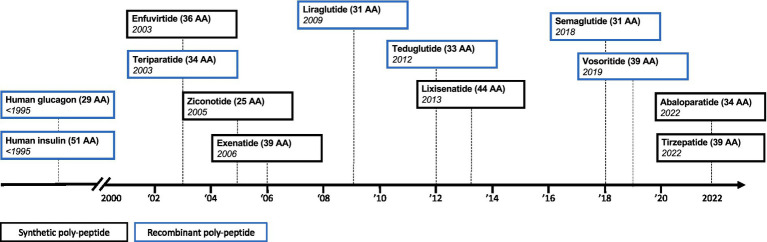
Timeline of synthetic and recombinant polypeptide approvals. AA = amino acid.

Synthetic drug manufacturing has yielded ever-more complex synthetic drug products for the European market (Figure 1). This has now resulted in a cost-effective approach to produce synthetic drug products that are similar in size and complexity to some recombinantly produced biologics. These advances in synthetic drug manufacturing have introduced a new dynamic to the regulatory field of complex generics, as we now witness chemically synthesized polypeptide products coming to the market, which use a biological originator product as their reference medicine.

### Ambiguity about regulatory approval of synthetic peptides

Stimulating the development and approval of generics and biosimilars plays a fundamental role in providing lower-cost therapeutically equivalent treatment options and hence reducing the financial burden on healthcare systems. At the same time, regulatory systems need to assure that only safe and high-quality generics and biosimilars enter the market.

To this end, the EMA has also noticed the recent developments in the field of chemically synthesized polypeptide products, emphasizing that from an analytical and regulatory perspective, specific considerations should apply to this class of therapeutics as they are at the interface between small molecules and biologics (3). However, while biosimilars are subject to robust analytical and clinical comparisons with their reference product through the dedicated biosimilar pathway, synthetically produced follow-on products are not eligible for assessment through this pathway, since they fall outside the definition of a biological substance. These products must therefore seek approval via traditional regulatory pathways for generics, such as the generic pathway under Article 10 (1), or via the hybrid pathway under Article 10 (3). The regulatory pathways for small-molecule generics typically involve less stringent regulatory requirements, for example regarding clinical assessment to demonstrate similarity, compared to their biosimilar counterparts. This could have potential implications for clinical practice with regard to documentation, prescribing, dispensing or the reporting of adverse drug reactions (ADRs). In this review, we describe the landscape of synthetic peptides referencing biological originators with examples from three case products: (i) liraglutide, (ii) human glucagon, and (iii) teriparatide.

### Examples of synthetic peptides referencing biological originator products

#### Liraglutide

In December 2022, a generic synthetical version of the reference biological liraglutide (Victoza^®^) has been developed. The product Viatide^®^ used the traditional generic pathway under Article 10 (1) but was refused marketing authorisation by the Danish Medicines Agency that functioned as the reference member state (RMS). The Danish authority indicated that the marketing authorization could not be granted due to *“major objections qualifying as potential serious risk to public health”* as the chemical-pharmaceutical documentation and Quality Overall Summary in relation to Viatide^®^ were not considered of sufficient quality in view of the present European regulatory requirements to recommend a marketing authorization (4). A critical issue regarding the fibrillation characteristics remained.

#### Human glucagon

Recombinant human glucagon (Glucagen^®^) has been approved for more than three decades. In February of 2021, Ogluo^®^, a synthetically produced follow-on version of the biological reference product Glucagen^®^ has been approved by EMA via a hybrid application procedure under Article 10 (3). This synthetic version is provided as pre-filled pens and pre-filled syringes whereas the biological originator is available as powder to be reconstituted. However, while the formulation differs between the two products, it concerns the same active substance (glucagon) and hence Ogluo^®^ was approved by EMA and considered bioequivalent to the originator product Glucagen^®^.

#### Teriparatide

In contrast to the liraglutide and glucagon case presented above, several manufacturers have developed biosimilars to the biological originator teriparatide (Forsteo^®^). In January of 2017 the first biosimilars gained approval in the EU: Terrosa^®^ and Movymia^®^, both manufactured by Richter-Helm BioLogics (5, 6). Due to their biological origin, these products were approved via the biosimilar pathway under Article 10 (4). Shortly after the approval of the first wave of biosimilars, the first synthetically produced teriparatide was approved in Europe in May of 2017. This synthetic version of teriparatide, manufactured by Teva, was approved via the hybrid pathway of Article 10 (3) (7). The European landscape of teriparatide has further evolved in the last two years with the biosimilar approvals of Livogiva^®^ in August 2020, as well as Sondelbay^®^ (March 2022) and Kauliv^®^ (January 2023), which are both developed by Indian-based manufacturers. However, teriparatide Cinnagen^®^, manufactured by Iranian manufacturer Cinnagen, recently failed to gain biosimilar approval in the EU. The Committee for Medicinal Products for Human Use (CHMP) expressed concerns about the quality of the product, including the nature of impurities and how the product was produced (8). These concerns made it impossible to conclude that Teriparatide Cinnagen was highly similar to the originator Forsteo^®^, which underlines the scientific challenges of developing high quality biosimilars.

Following the five biosimilar approvals and Teva’s approval of a synthetically produced generic version of teriparatide via the hybrid pathway, two more synthetically produced generic versions of teriparatide have recently been approved in the EU through the traditional generic pathway of Article 10 (1): Teriparatide Welding^®^, manufactured by the Spanish company GP-Pharm (August 2021), and Teriparatide Ambio^®^, manufactured by the French company Viatris Santé (March 2022). The generic pathway under Article 10 (1) that was used in this case did not require submitting additional clinical data. Interestingly however, whereas Teriparatide Welding^®^ submitted a generic application without any clinical bioequivalence studies, Teriparatide Ambio^®^ did submit a bioequivalence study (9, 10).

**Figure 2:**
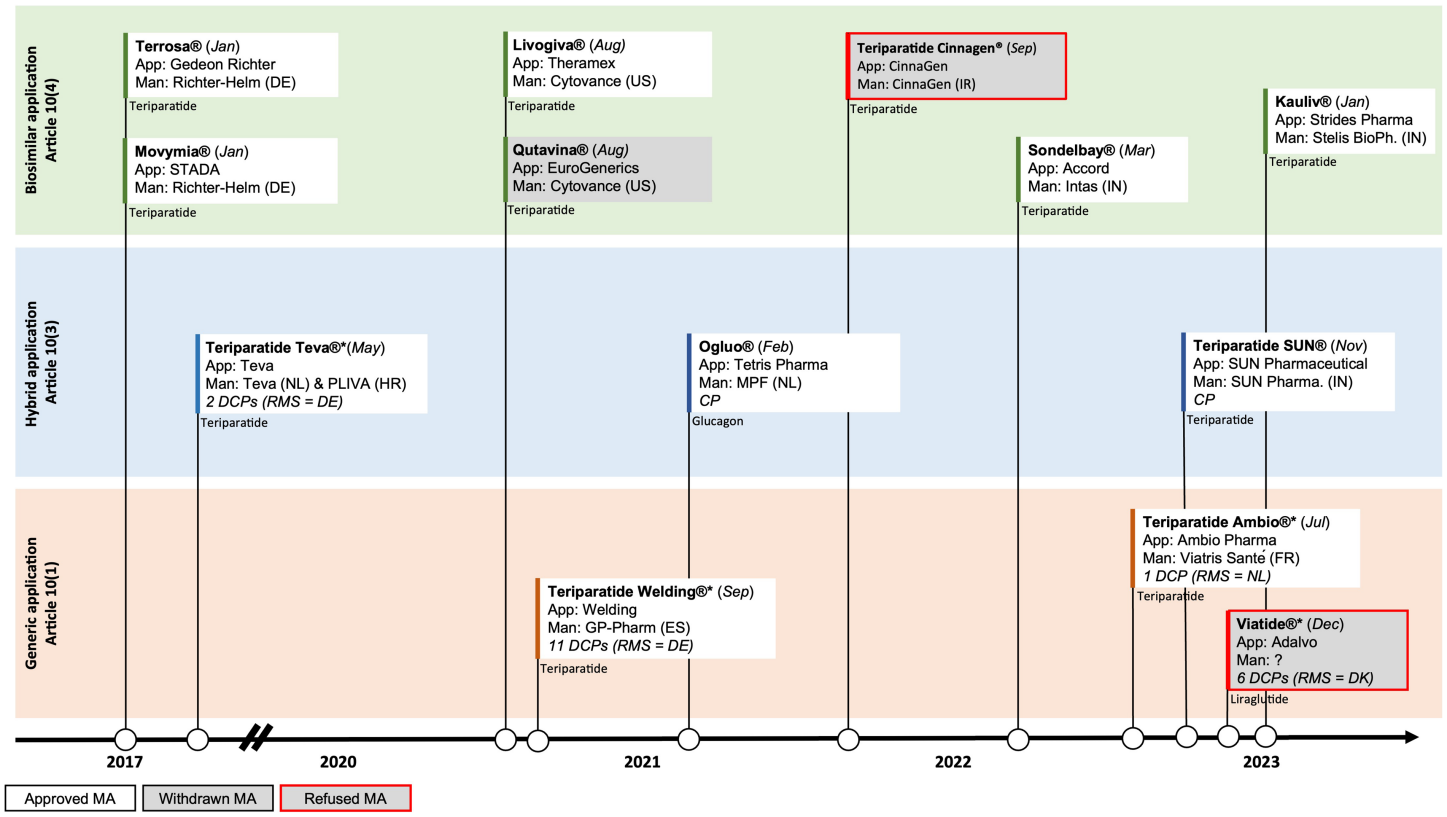
Timeline of EU approvals of polypeptide follow-on products and the abridged applications used for (i) liraglutide, (ii) glucagon, and (iii) teriparatide; App = Applicant, Man = Manufacturer, RMS = Reference Member State, CP=Centralised Procedure, DCP=Decentralised Procedure; DE = Germany, DK=Denmark, ES=Spain, FR = France, HR = Croatia, IN=India, IR = Iran, NL = The Netherlands, US=United States of America.

Teva, Welding and Ambio’s products are all approved via the decentralised procedure (DCP), relying on assessments by different Member States. Whereas Teva and Welding chose Germany as their RMS, Ambio chose the Netherlands as RMS (Figure 2). The latest approval of a synthetic version of teriparatide by SUN Pharmaceuticals (November 2022) however went through the hybrid pathway under Article 10 (3) of the centralised procedure (CP) by EMA (11). This now totals to four synthetically produced generic approvals via different regulatory pathways (generic and hybrid) and procedures (DCP and CP), and using different regulatory bodies [BfArM (Germany), MEB (the Netherlands) and EMA], with also another five biosimilar versions of teriparatide on the market.

### Potential implications of inconsistent regulatory approaches

Although both recombinant and synthetic versions of polypeptide medicines have the same active ingredient and treat the same condition, they fall within two different regulatory frameworks. This can have important implications for regulatory assessments, clinical practice and pharmacovigilance.

#### Divergence of regulatory assessments with DCPs and different RMSs

Synthetically produced teriparatide products made use of DCPs in contrast to the mandatory CP through EMA for recombinant biologics and hence teriparatide biosimilars. The synthetic teriparatide applications by Teva, Welding, Ambio and SUN Pharmaceutical made use of different RMSs or EMA. This may create challenges for ensuring consistency in regulatory assessments. This is not unique to synthetic peptides referencing biologics, as similar challenges has been described for so-called non-biological complex drugs (NBCDs) and it could be potentially more precarious for more complex polypeptide products where comparison by physicochemical analytical methods alone may not be sufficient (12–14). The extent and practical implications of heterogenic regulatory approaches are currently unknown, and EMA has only recently issued a draft guideline on the development and manufacture of synthetic peptides using a biologic as a reference medicinal product (3). EMA has systems in place to stimulate uniform evaluations under the DCP, such as referral procedures to the CHMP to solve any disagreements among Member States. Nonetheless, the recent developments in this field call for more consideration among European regulatory bodies to create a harmonised regulatory approach adapted to the inherent complexities of this novel class of generics (15–17).

#### Pharmacovigilance and traceability of products

Due to their complexity, biologics are subject to specific pharmacovigilance requirements, mandating the identification of the brand name and batch number in adverse drug reaction (ADR) reporting (18). However, since the generic applications by Teva, Welding, Ambio, and SUN Pharmaceutical concern synthetic products, these products fall outside of the scope of this part of the Directive. This could hamper and potentially delay the identification of new safety issues for specific products. On the one hand, generics approved via the generic procedure of Article 10 (1) or the hybrid procedure of Article 10 (3) can be approved under the generic name only, which limits the ability to identify product specific safety issues. On the other hand, generics approved via the DCP can be approved under a variety of different brand names and marketing authorisation holders in different EU Member States, because the use of the DCP does not require the use of a single brand name throughout the European market (which is the case for approval via the CP). For example, Welding’s synthetic teriparatide makes use of 11 DCPs, each involving a unique brand name for use in different Member States. This could further place a burden on the timely identification of product or batch specific safety signals, for example in pan-European safety databases such as EudraVigilance. Therefore, requirements with regard to documentation and reporting of ADRs (e.g., brand name traceability) should also be applied to all synthetically produced generics referencing biological originator products, to make sure that these products fulfil the same regulatory standards.

#### Clinical practice and pharmacy-mediated substitution

The use of the traditional generic pathway under Article 10 (1) could also lead to different practices regarding pharmacy-mediated substitution and switching between products. For biosimilars this is generally under the prescriber’s supervision and closely monitored. In contrast, for ‘traditional’ generics pharmacy-mediated substitution can be done at the retail pharmacy level in many jurisdictions. The presence of synthetic and biologically produced versions of the same active substance may therefore create additional challenges for clinical practice when it comes to guidelines for switching between originator, biosimilar and synthetic versions. It is consequently of particular importance to ensure that any switching between different versions is closely monitored, especially when synthetically-produced and recombinantly-produced versions of the same product coexist in national markets. This is further complicated by the fact that some Member States presume therapeutic equivalence for generic approvals via Article 10 (1), which could result in pharmacy-mediated substitution (e.g., switching from rDNA to synthetic version of teriparatide) without supervision or appropriate documentation. The latter becomes even more important for pharmacovigilance, which plays a critical role in ensuring safety of generic entries by detecting any potential safety issues in routine clinical practice.

### Final considerations

Polypeptides and smaller proteins are generally considered to pose lesser concerns with regards to immunogenicity than larger proteins. However, synthetic follow-on products referencing a biologically produced polypeptide product may exhibit a different impurity profile compared to the reference product with potential implications for immunogenicity (14). This could call for additional studies, and therefore the hybrid pathway under Article 10 (3) may be considered more appropriate than the traditional generic pathway under Article 10 (1) for these type of products. Although the practical implications of potential heterogenic regulatory approaches are still unknown and it is outside the scope of this article to evaluate the quality of the regulatory assessment of the products approved so far, we advocate for a consistent regulatory approach for approving complex generics. European legislators have determined that medicines with a certain level of complexity can only be licensed via the CP. These include recombinant biologics (including biosimilars), advanced-therapy medicinal products, orphan medicines, and medicines to treat a number of defined diseases/disease classes. EMA acknowledges that the basic principles for biosimilars should also be considered for synthetic polypeptides referencing a biologic (3). Consequently, it can be considered to confine these products to the CP, like recombinant biologics and their biosimilars. The benefit of the CP is that it leads to a single evaluation, with expert involvement from all EU Member States, which can ensure consistency of regulatory assessments, as well as a single marketing authorisation throughout the EU, which can address challenges related to pharmacovigilance and traceability.

Although the case examples above focus on the EU setting, the matter in question is applicable globally. For example, in Canada and South Korea biosimilar and synthetic teriparatide versions also co-exist in national markets. Moreover, CinnaGen’s teriparatide biosimilar which has been denied marketing authorisation in the EU, is marketed in Iran since 2013. In the US, FDA has recently implemented guidance on appropriate use of the 505(j) pathway for synthetic peptides referencing medicines of rDNA origin, which is the US’ equivalent of the EU’s generic pathway of Article 10 (1) (19). This is expected to open the avenue for approving synthetically produced peptide products. With the rapid advances in synthetic drug manufacturing seen over the past years, more complex synthetic products referencing recombinant originator products are expected in the coming years. Global alignment of regulatory approaches is therefore crucial to ensure that only safe and high-quality follow-on products reach patients in global markets. Future research and close monitoring of this fast-developing product class of polypeptide medicines should help to inform on current knowledge gaps and potential impact on public health.

## Author contributions

KK: Conceptualization, Investigation, Writing – original draft, Writing – review & editing. JH: Writing – review & editing. PS: Writing – review & editing.
